# Comparative analysis of methods for measuring glucocerebrosidase enzyme activity in patients with Parkinson’s disease with the *GBA1* variant

**DOI:** 10.3389/fneur.2025.1523655

**Published:** 2025-04-02

**Authors:** Jin Hwangbo, Myung Jun Lee, Sang Jin Kim, Hyun Kyung Park, Jae-Hyeok Lee

**Affiliations:** ^1^Busan St. Mary's Hospital, Busan, Republic of Korea; ^2^Pusan National University Hospital, Busan, Republic of Korea; ^3^School of Medicine, Pusan National University, Yangsan, Republic of Korea; ^4^Inje University Busan Paik Hospital, Busan, Republic of Korea; ^5^Special Chemistry Team, Seoul Clinical Laboratories (SCL), Yongin, Republic of Korea; ^6^Pusan National University Yangsan Hospital, Yangsan, Republic of Korea

**Keywords:** Parkinson’s disease, *GBA1*, glucocerebrosidase, blood biomarkers, glycosphingolipids

## Abstract

**Introduction:**

*GBA1* variants are significant genetic risk factors for Parkinson’s disease (PD). Accurately measuring glucocerebrosidase (GCase) activity is crucial for understanding disease progression and developing targeted therapies. This study aimed to validate strategies for measuring blood GCase activity in patients with *GBA1*-associated PD (GBA-PD).

**Methods:**

We recruited 25 GBA-PD patients and 27 matched PD patients without *GBA1* variants (non-GBA-PD). GCase activity from fresh blood was quantified using the 4-methylumbelliferyl *β*-D-glucopyranoside leukocyte assay (GCaseRaw). The GCase patient/normal control ratio (GCase ratio) was calculated for consistency. GCase activity in dried blood spot (DBS) specimens (GCaseDBS) and plasma glucosylsphingosine (GluSph) levels were measured using LC–MS/MS. The diagnostic accuracy was assessed using area under the curve (AUC) values.

**Results:**

No significant differences in demographics or disease characteristics were found between GBA-PD and non-GBA-PD patients. GCase activity was significantly lower in patients with GBA-PD (*p* < 0.001). The GCase ratio exhibited a higher diagnostic accuracy (AUC, 0.93) than GCaseRaw (AUC, 0.88) or GCaseDBS (AUC, 0.78). Plasma GluSph levels were higher in GBA-PD patients and were negatively correlated with the GCase ratio (*r* = −0.326; *p* < 0.01).

**Discussion:**

The relative ratio of GCase activity showed a strong discriminatory potential, distinguishing between GBA-PD and non-GBA-PD.

## Introduction

Variants in *GBA1*, which encodes the lysosomal enzyme *β*-glucocerebrosidase (GCase), are among the most significant genetic risk factors for Parkinson’s disease (PD). Studies have found that heterozygous mutations in *GBA1* can lead to decreased GCase activity, resulting in the accumulation of glycosphingolipids, which are believed to contribute to the pathogenesis of PD by impairing autophagic processes and promoting the aggregation of *α*-synuclein (1).

Based on their role in Gaucher’s disease (GD), *GBA1* variants are classified as ‘severe’ (e.g., p.L483P, previously known as L444P) or ‘mild’ (e.g., p.N409S, N370S) (2). ‘Risk’ variants (e.g., p.E365K, E326K) are associated with PD risk but do not cause Gaucher’s disease. Severe *GBA1* variants are associated with a higher risk of PD, younger onset, and more rapid disease progression, whereas mild and high-risk variants are associated with a more benign course (1, 2). The severity of *GBA1* variants is known to affect functional biomarker profiles in patients with *GBA1*-associated PD (GBA-PD) (3, 4). Severe variants showed the lowest GCase levels with the steepest decline over time, as well as the lowest CSF total alpha-synuclein and highest seeding activity, indicating more aggressive pathology (5, 6).

GCase activity is generally reduced in peripheral blood samples from patients with GBA-PD, accompanied by the accumulation of substrates, similar to the reductions observed in the brain and CSF (3). The two key substrates of GCase are glucosylceramide (GluCer) and glucosylsphingosine (GluSph) (7). GlcSph is a more clinically useful biomarker, as it correlates with disease burden in GD. Accurate measurement of GCase activity is crucial for understanding the biochemical impact of *GBA1* variants and for developing targeted therapies to enhance GCase function in patients with GBA-PD. However, considerable variability in the methods used to measure GCase activity in disease models and patient populations may impede the development of effective GCase therapies (8, 9). This study aimed to validate widely used strategies for measuring GCase activity in blood samples from patients with GBA-PD to assess their diagnostic efficacy and correlation with GlcSph levels.

## Materials and methods

We recruited 25 patients with GBA-PD and 27 disease duration-matched patients with PD without *GBA1* variants (non-GBA-PD). All patients fulfilled the Movement Disorder Society’s diagnostic criteria for PD (10). The study was approved by the Institutional Review Board of Pusan National University Yangsan Hospital (No. 05–2023-189), and informed consent was obtained in accordance with the recommendations of the Declaration of Helsinki. To detect *GBA1* variants, the entire *GBA* gene, including all 11 exons and intron-exon boundaries, was sequenced using a long-range polymerase chain reaction approach to exclude the amplification of its pseudogene (11). GBA mutations were annotated according to NM_000157.4 and NP_000148.2. Immediately after blood collection, whole-blood and dried blood spot (DBS) samples were sent to a commercial testing laboratory for comprehensive analysis. GCase activity (nmol/h/mg) from fresh blood was quantified using the 4-methylumbelliferyl *β*-D-glucopyranoside (4-MUG) leukocyte assay (GCaseRaw), corrected for white blood cell counts. The GCase activities of each patient and three controls (blank, normal control, and abnormal control) were measured simultaneously. The GCase patient/normal control ratio (%) was also calculated for consistency (GCaseRatio) (11). GCase activity (μmol/h/L) from DBS specimens (GCaseDBS) and plasma glucosylsphingosine (GluSph, ng/mL) concentrations were measured using liquid chromatography with tandem mass spectrometry (LC–MS/MS). Further experimental details are provided in the Supplementary material.

The data processing and statistical analyses were performed using SPSS (version 29.0.2). Group differences were analyzed using the Mann–Whitney *U* test for continuous variables and the *χ*^2^ test for categorical variables. Age and disease duration were controlled using an analysis of covariance. Diagnostic accuracy was assessed using the corresponding area under the curve (AUC) values. The figures were generated using Python 3.12.5 with the matplotlib and Seaborn libraries.

## Results

**Figure 1:**
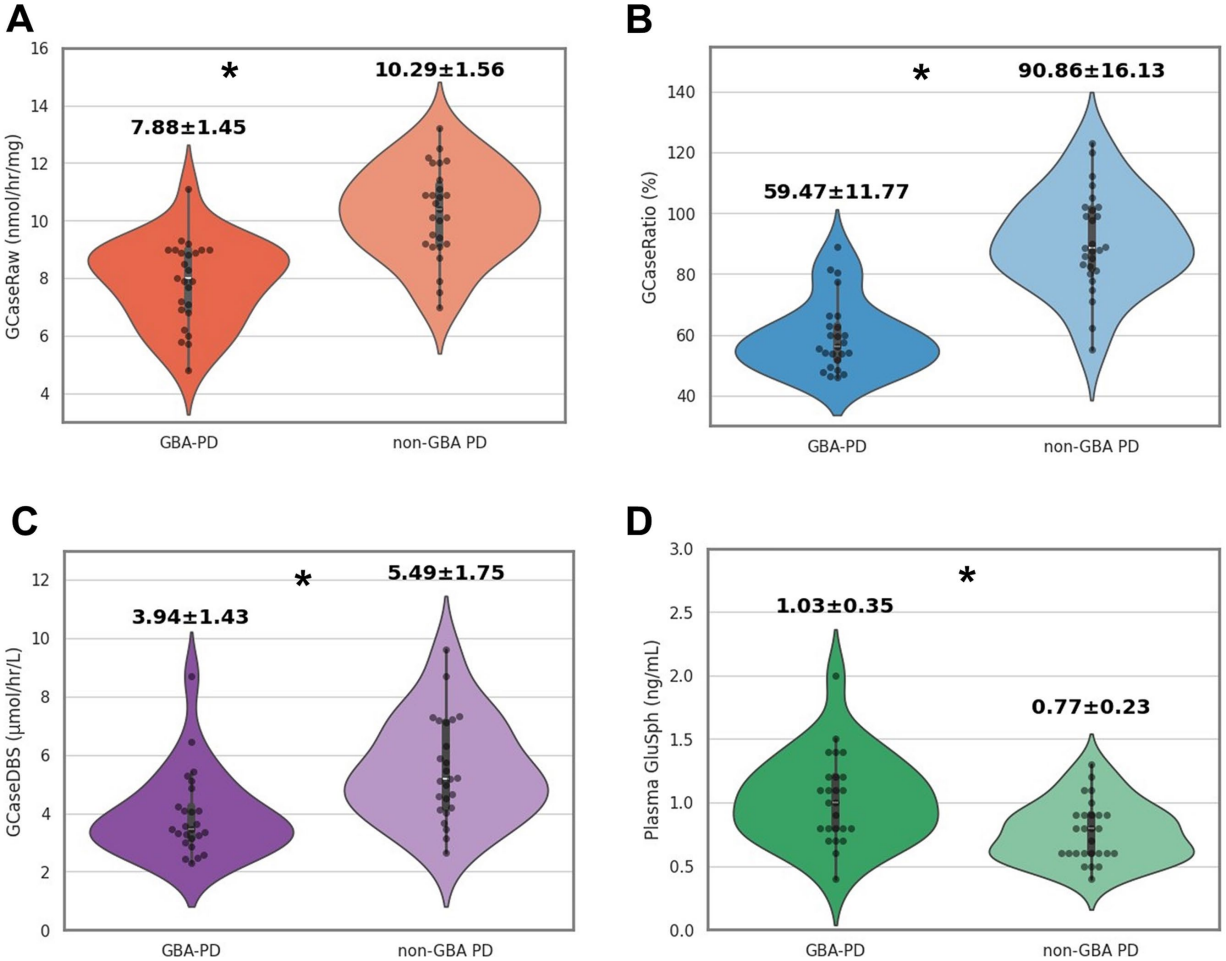
Comparison of GCase activity and diagnostic accuracy between GBA-PD and non-GBA PD patients. **(A)** GCaseRaw (nmol/h/mg): GCase activity measured in fresh blood. **(B)** GCaseRatio (%): the ratio of GCase activity in patients relative to healthy controls. **(C)** GCaseDBS (μmol/h/L): GCase activity measured in dried blood spot (DBS) samples. **(D)** Plasma GluSph (ng/mL): The concentration of GluSph measured in plasma. *p < 0.001.

**Figure 2:**
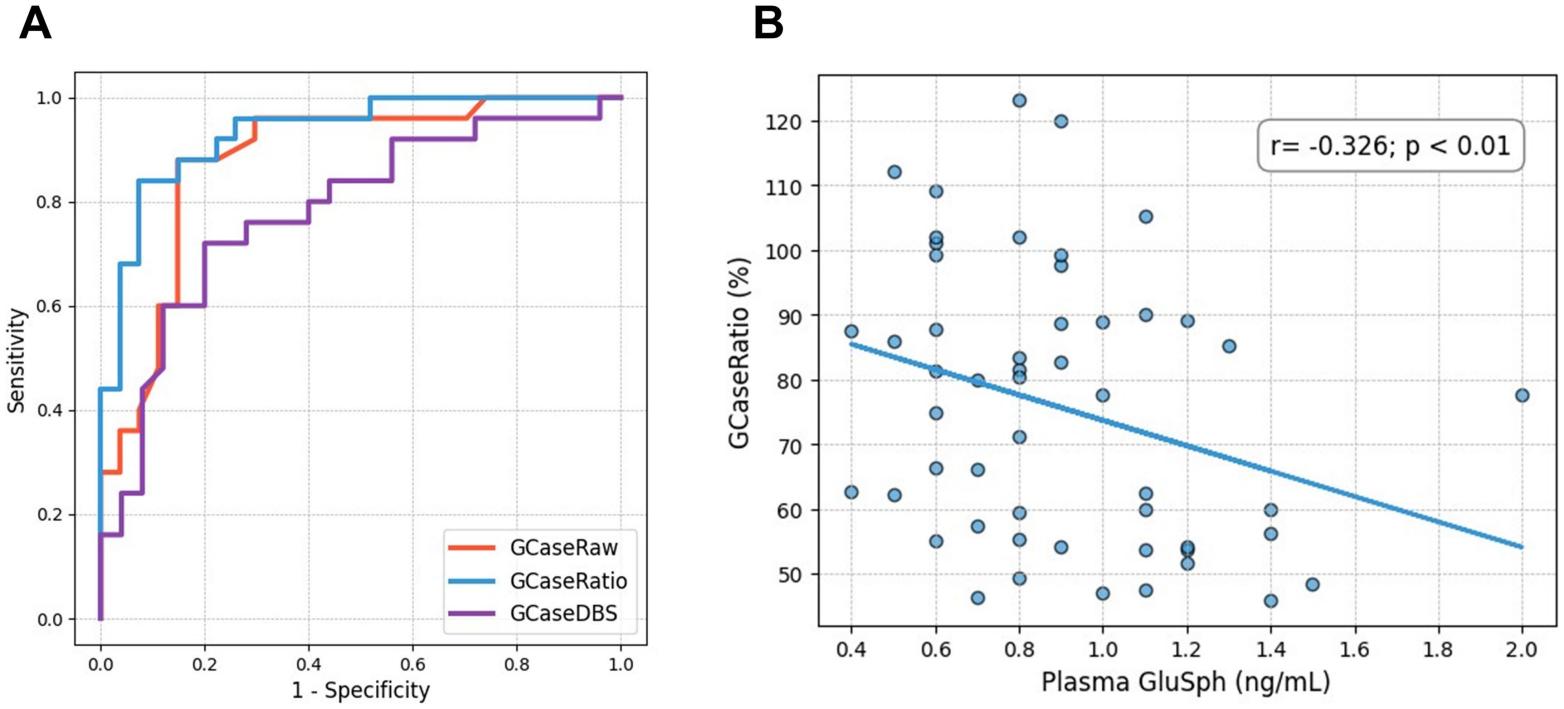
**(A)** Area under the curve (AUC) for GCaseRaw, GCaseRatio, and GCaseDBS. **(B)** A scatter plot shows the negative correlation between GluSph and GCaseRatio.

The clinical features of patients with GBA-PD and non-GBA-PD are presented in Table 1. There were no significant differences between GBA-PD and non-GBA-PD patients in age at onset, age at evaluation, disease duration, sex, Unified Parkinson’s Disease Rating Scale Part III (UPDRS-III) scores, Hoehn and Yahr stage, Mini-Mental State Examination (MMSE), Montreal Cognitive Assessment (MoCA) performance, and levodopa-equivalent daily dose (LEDD). The heterozygous *GBA1* variants identified among the patients included p.L483P (*n* = 8), p.R159W (R120W, *n* = 3), p.N227S (N188S, *n* = 2), p.F252I (F213I, *n* = 2), p.D448H (D409H, *n* = 2), p.R202* (R163*, *n* = 1), p.I442V (n = 1), c.115 + 1G > A (*n* = 1), p.N431S (N392S, *n* = 1), Rec1 [p.L483P; p. A495P; p.V499=] (*n* = 1), p.G416S (G377S, *n* = 1), p.V211fs (c.630delC, *n* = 1), and p.G85E (G46E, *n* = 1). Most of these patients (21 of 25) were classified as having severe variants (2). Among the remaining four *GBA1* variants, p.G85E was classified as mild, while p.I442V, p.N431S, and p.V211fs were categorized as unknown. GCase activity from all specimens was significantly reduced in GBA-PD patients (*p* < 0.001), compared to non-GBA-PD patients (GCaseRaw, 7.88 ± 1.45 nmol/h/mg vs. 10.29 ± 1.56 nmol/h/mg, *p* < 0.001; GCaseRatio, 59.47 ± 11.77% vs. 90.86 ± 16.13%, *p* < 0.001; GCaseDBS, 3.94 ± 1.43 μmol/h/L vs. 5.49 ± 1.75 μmol/h/L, *p* < 0.001) (Figures 1A–C). The GCaseRatio exhibited higher diagnostic accuracy (AUC, 0.93; 95% confidence interval [CI], 0.86–0.99) than GCaseRaw (AUC, 0.88; 95% CI, 0.78–0.96) or GCaseDBS (AUC, 0.78; 95% CI, 0.64–0.91) (Figure 2A). Plasma GluSph concentration was significantly higher in GBA-PD patients than in non-GBA-PD patients (1.03 ± 0.35 ng/mL vs. 0.77 ± 0.23 ng/mL, *p* < 0.001) (Figure 1D) and was negatively correlated only with GCaseRatio (*r* = −0.326; *p* < 0.01) (Figure 2B). There were no associations between blood biomarkers (GCase activity and plasma GluSph levels) or clinical measures (UPDRS-III, H-Y stage, MMSE, and MoCA).

**Table 1:** Demographic and clinical characteristics of GBA-PDA and non-GBA-PD.

	GBA carriers (*n* = 25)	GBA non-carriers (*n* = 27)	*p* value
Age of onset (yr)	52.3 ± 9.3	47.7 ± 7.5	0.078
Age at evaluation (yr)	57.4 ± 8.4	53.2 ± 7.4	0.08
Disease duration (yr)	4.6 ± 3.1	4.8 ± 2.7	0.719
Sex (Male/female)	11/14	13/14	0.773
UPDRS-III	25.8 ± 8.5	24.4 ± 17.9	0.135
Hoehn & Yahr	2.0 (1.0 ~ 2.5)	2.0 (1.0 ~ 2.5)	0.518
MMSE	27.6 ± 2.2	28.7 ± 1.5	0.071
MoCA	25.0 ± 4.4	26.8 ± 3.1	0.088
LEDD (mg)	612.6 ± 546.8	612.2 ± 379.5	0.318

## Discussion

Our study revealed significant alterations in the blood GCase activity and GlcSph levels in patients with GBA-PD, characterized by decreased GCase activity and elevated GluSph levels. The differences between GBA-PD and non-GBA-PD were more distinct in our study than in previous research, possibly because our cohort had a higher proportion of severe variants and a markedly lower prevalence of risk variants than Western populations. Previous studies have shown that severe variants exhibit lower GCase activity levels and demonstrate a more significant decline over time during follow-up (3, 4).

GCase activity can be measured in various samples, including leukocytes, DBSs, and cultured cells (8, 12). Our comparative analysis demonstrated that the 4-MUG leukocyte assay had better diagnostic accuracy than the mass spectrometer-based method for DBS samples. This result was expected, given its value for diagnosing GD (12). DBS samples are useful for screening clinically suspected individuals and offer advantages such as ease of collection, small blood volume requirements, and simple transportation and storage. The 4-MUG leukocyte assay, with its established specificity and sensitivity, is the gold standard for measuring GCase activity (12).

Recent studies have used DBS to assess GCase activity in large cohorts of patients (4, 13–15). However, the correlation between *GBA1* variant severity and GCase activity varies across studies. The DBS assay measures the enzymatic activity in protein lysates, including cytosolic and lysosomal GCase (8). Factors such as blood volume, hematocrit, and pre-analytical steps such as drying time can also affect the results (12). Therefore, heterozygotes may have half-normal enzyme activity, overlapping with healthy controls, making enzyme determination in DBS samples for carrier status less reliable.

The diagnostic accuracy of the 4-MUG leukocyte assay significantly improved. The relative ratio of GCase activity, calculated for consistency, showed enhanced discriminatory potential, distinguishing between the GBA-PD and non-GBA-PD groups. Moreover, this method revealed an inverse association between GCase activity and its substrate, plasma GluSph, a valuable biomarker for monitoring and modulating the efficacy of interventions to increase GCase activity (7, 13).

Despite its contributions, this study had several limitations. The small sample size may restrict the generalizability of our findings. We were unable to identify an association between blood biomarkers and PD status in the current samples. Unlike previous reports (1, 2), severe variants in our cohort did not show worse clinical features, possibly due to the short disease duration. Future research with larger cohorts, including a diverse range of variant severities, is warranted to establish the reliability of these blood biomarkers in clinical settings and to investigate the variability in GCase activity across different populations and stages of PD.

## Data Availability

The original contributions presented in the study are included in the article/supplementary material, further inquiries can be directed to the corresponding author.
