# Supplementary material for: Comparative analysis of methods for measuring glucocerebrosidase enzyme activity in patients with Parkinson’s disease with the GBA1 variant
Source: Front Neurol. 2025 Apr 2;16:1523655. doi: 10.3389/fneur.2025.1523655 (PMC11999970; doi:10.3389/fneur.2025.1523655)
Supplement: Supplementary file 1 [file Data_Sheet_1.PDF]

GCase Activity Measurement

1. Fluorometry Assay (Leukocyte-based method)

- Substrate: 5.02 mM 4-methylumbelliferyl β-D-glucopyranoside in 0.2 M phosphate-citrate buffer (pH 5.4).
- β-Glucocerebrosidase hydrolyzes this substrate, releasing fluorescent 4-Methylumbelliferone, which reflects enzyme activity.
- Quality control was performed at a minimum of two levels (normal/abnormal), as recommended. However, as commercial quality control materials are not available for this assay, previously tested patient samples were used. Each batch included blank, normal control, and abnormal control samples, which were analyzed alongside patient samples. The blank sample was included to confirm the absence of non-specific reactions.
- The normal control sample was not collected at the same time as the patient samples. Instead, previously validated normal samples from earlier batches were used. Sample stability was maintained for up to one month at -20°C, and only normal samples within this timeframe (typically within one week) were used. If no suitable normal control was available, a fresh sample was processed, cells were isolated, and GCase activity was measured to confirm normal levels before use as a normal control.
- While β-glucosidase inhibitors like conduritol B-epoxide can enhance assay specificity, their necessity depends on assay validation and intended application. In this study, we followed an established commercial protocol that does not include conduritol B-epoxide validation.

2. LC-MS/MS (DBS-based method)

- A 3.2 mm DBS punch was incubated with a manufacturer-provided β-glucocerebrosidase-specific substrate, using the NeoLSD MSMS kit, a commercial kit exclusively provided by PerkinElmer, and the resulting product was quantified via LC-MS/MS.
- According to the kit insert, the substrate dissolution reagent has a pH of 4.7.
- Enzyme activity was expressed as μmol/h/L based on peak ratios of the product and an internal standard.

GlcSph Measurement via LC-MS/MS

- Sample preparation: plasma (100 μL) extracted with chloroform:methanol (2:3, v/v, 1 mL), centrifuged, dried, and reconstituted in 200 μL of 90% acetonitrile before filtration and injection (2 μL).
- LC-MS/MS Conditions:
  - Column: Unison UK C18 (2.0 × 50 mm, 3 μm, Imtakt, USA).
  - Mobile phase: A (0.1% formic acid in water) / B (0.1% formic acid in acetonitrile).
  - Mass spectrometry (MRM transitions, ESI positive ion mode):
    - GlcSph: m/z 462.216 → 282.200
    - Internal standard (N, N-Dimethylsphingosine): m/z 328.249 → 310.200
